# Electrochemical Enzyme Immunoassay

**DOI:** 10.6028/jres.093.131

**Published:** 1988-06-01

**Authors:** H. Brian Halsall, William R. Heineman, Sarah H. Jenkins, Kenneth R. Wehmeyer, Matthew J. Doyle, D. Scott Wright

**Affiliations:** Analytical and Biochemistry Divisions, and Biomedical Chemistry Research Center, Department of Chemistry, University of Cincinnati, Cincinnati, OH 45221-0172

“Immunoassay” defines the body of techniques which use the antibody (Ab) macromolecule, usually of the IgG class, for the detection and quantitation of an enormous range of simple and complex antigen (Ag) molecules. The success of the methods relies on both the specificity and formation constant of the Ab used, and the ability to detect the interaction between Ab and Ag. Sensitive assays in complex matrices require some kind of label to be present to provide this ability.

The development of immunoassay methods has in large part been driven by the available technology. Thus, although one of the first immunoassays using a label was electrochemically based [1], the state of that art in 1951 was relatively primitive, and could not meet the analytical demands of the Ag. Some years later, Yalow and Berson [2] developed immunoassays based on the radioisotopic label (RIA). For the first time, Ab selectivity was married to very low detection limit technology, and the result was an enormous growth rate in the use of RIA in both the clinical and research laboratory.

RIA has the significant advantage that the label used is not a normal constituent of physiological samples, and interferences of this type are therefore absent. RIA, however, has the disadvantages that accompany radioisotope handling, together with an inability to distinguish label which is bound from that which is not. There has therefore been an enormous effort to find suitable replacements for the radiolabel.

Where the low detection limit of RIA was not required for a successful assay, labels detectable by spectroscopic methods have become important. Where very low detection limits are demanded, then the concept of amplification of, or by, the label has been developed. The most successful of these have been enzyme linked using an immunosorbent phase for Ag extraction (ELISA), although assays based on the lysis of label-containing liposomes are also exhibiting very low detection limits.

In our hands, electrochemically based immunoassays at very low detection limits have also used enzyme amplification and ELISA, with either NAD/NADH and glucose-6-phosphate dehydrogenase [3], or phenyl phosphate/phenol and alkaline phosphatase [4]. The greatest sensitivity thus far has been obtained with ELISA coupled with liquid chromatography with electrochemical detection (LCED) [5].

The power of this approach can be readily illustrated by examining the evolution of its use in an assay for IgG, itself an important analyte. This evolution covers three principal stages of development and a reduction in detection limit of five orders of magnitude.

The basic methodology is shown in figure 1. The assay is essentially a “sandwich” ELISA, using an alkaline phosphatase labelled second Ab for amplification. Oxidative flow amperometry at +875 mV on a carbon paste electrode is used for quantitation of the phenol produced. The phenyl phosphate substrate is electroinactive at this potential, and does not interfere. All analyses were conducted using a 20 μL injection loop for LCEC.

The first stage of development used a polystyrene cuvette of 750 μL as the solid phase to which the primary Ab was passively adsorbed, and Tween 20 as a blocking agent to minimize nonspecific adsorption of reagents to the plastic surface. Under optimal conditions, a detection limit of 500 attomoles of analyte IgG (or 300 million molecules) was obtained [6]. However, not counting the primary Ab adsorption step, this was a lengthy assay, requiring some 7 hours for completion.

The second stage of development was directed at reducing the level of the nonspecific background. This was achieved by incorporating bovine serum albumin in all solutions except that used for the adsorption of the primary Ab to the solid phase. This provides for more efficient blocking of the exposed surface, and prevents the adsorption of the second Ab-conjugate, which is the primary source of nonspecific signal [5]. Figure 2 shows the calibration curve for this stage, with a detection limit of 50 attomoles, or 30 million molecules [5] for exactly the same incubation conditions as for stage one. The length of the assay was not affected, therefore, remaining at 7 hours.

The third stage incorporated two major changes involving the nature of the solid surface and the attachment of the primary Ab to it. Since the concentration of phenol produced by unit activity of enzyme depends upon the volume of the container, whereas the amount produced does not, then increasing the surface area to volume ratio of the container should dramatically reduce the time needed for detectable quantities of phenol to be produced. This will be enhanced by a change in container geometry that reduces the diffusion path-length for substrate. Reducing the diffusion path-length will similarly shorten the time required for both the Ag and conjugate incubation steps.

Both of these characteristics were provided by using glass microcapillary hematocrit tubes of 70 μL volume. The primary Ab solid phase was prepared by covalently attaching the Ab to a polymer deposited on the glass surface. As in stage two, both bovine serum albumin and Tween 20 were used as blocking agents in all solutions except for the primary Ab attachment. A simple modification of the injection port permitted the direct displacement of the contents of the capillary into the injection loop. Figure 3 shows the calibration curve for this assay, for which the detection limit was 4×l0^−3^ attomoles, or 2800±150 molecules. The total time of the assay, again after preparation of the primary Ab surface, was 28 minutes.

The design of the electrochemical flow cell in use permits a further reduction in its volume. By then reducing the volume of sample in the capillary, the limit of detectable amount should be readily lowered by an order of magnitude with no increase in assay time.

## Figures and Tables

**Figure 1:**
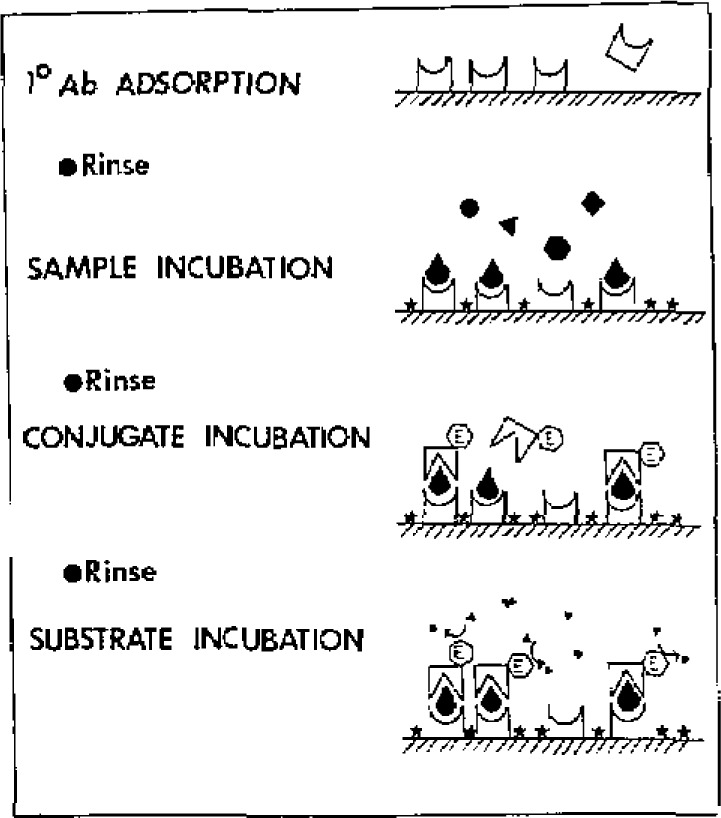
Heterogeneous assay protocol. Small stars represent blocking reagents (Tween 20 and bovine serum albumin) to minimize nonspecific adsorption of conjugate.

**Figure 2:**
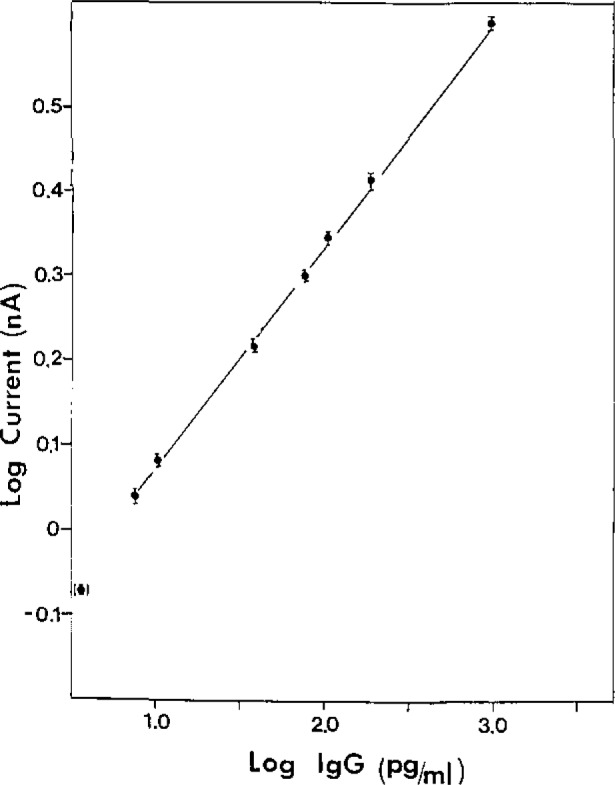
Calibration curve for Stage 2 assay in polystyrene cuvettes of 750 μL.

**Figure 3:**
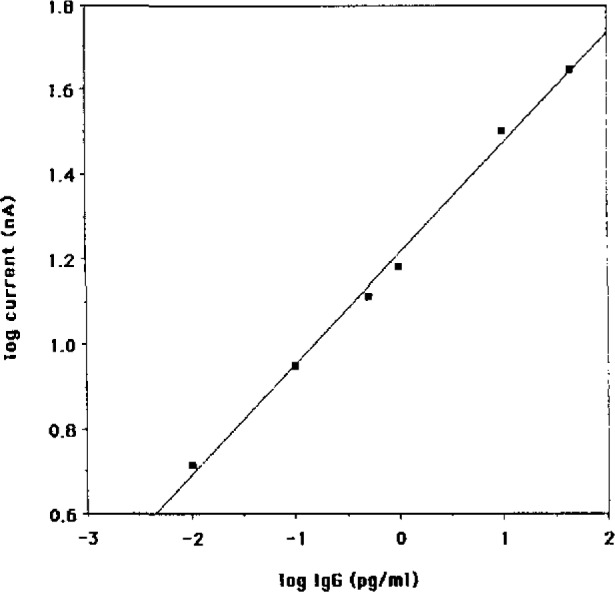
Calibration curve for Stage 3 assay in glass capillaries of 70 μL.
